# RETRACTION: Effect of Wound Drainage on the Wound Infection and Healing in Patients Undergoing Spinal Surgery: A Meta‐Analysis

**DOI:** 10.1111/iwj.70447

**Published:** 2025-03-26

## Abstract

**RETRACTION:** YangH. , BaoL. , LiJ. , WangY. , and YangJ. , “Effect of Wound Drainage on the Wound Infection and Healing in Patients Undergoing Spinal Surgery: A Meta‐Analysis,” International Wound Journal 21, no. 2 (2024): e14778, 10.1111/iwj.14778.38356179 PMC10867381

The above article, published online on 14 February 2024, in Wiley Online Library (http://onlinelibrary.wiley.com/), has been retracted by agreement between the journal Editor in Chief, Professor Keith Harding; and John Wiley & Sons Ltd. Following an investigation by the publisher, all parties have concluded that this article was accepted solely on the basis of a compromised peer review process. The editors have therefore decided to retract the article. The authors did not respond to our notice regarding the retraction.

**RETRACTION:** H. Yang , L. Bao , J. Li , Y. Wang , and J. Yang , “Effect of Wound Drainage on the Wound Infection and Healing in Patients Undergoing Spinal Surgery: A Meta‐Analysis,” International Wound Journal 21, no. 2 (2024): e14778, 10.1111/iwj.14778.38356179 PMC10867381

The above article, published online on 14 February 2024, in Wiley Online Library (http://onlinelibrary.wiley.com/), has been retracted by agreement between the journal Editor in Chief, Professor Keith Harding; and John Wiley & Sons Ltd. Following an investigation by the publisher, all parties have concluded that this article was accepted solely on the basis of a compromised peer review process. The editors have therefore decided to retract the article. The authors did not respond to our notice regarding the retraction.

